# Anti-Tumor Effects of *Vespa bicolor* Venom on Liver Cancer: In Vitro and In Vivo Studies

**DOI:** 10.3390/toxins17010004

**Published:** 2024-12-25

**Authors:** Yong-Hua Wu, Feng Xiong, Zheng-Wen Ou, Jing-An Wang, Jing Cui, Lin Jiang, Wen-Jian Lan

**Affiliations:** 1 School of Pharmaceutical Sciences, Sun Yat-sen University, Guangzhou 510006, China; wuyh276@mail2.sysu.edu.cn (Y.-H.W.); xiongf35@mail.sysu.edu.cn (F.X.); 2 Production and Research Base for Wasp Deinsectization, Huxin Biotechnology Co., Ltd., Jiangmen 529245, China; 1ozw@163.com (Z.-W.O.); kefu@hufenghuang.com (J.-A.W.); m18985140962@163.com (J.C.)

**Keywords:** *V. bicolor* Fabricius, venom, anti-tumor evaluation, monotherapy, combination therapy

## Abstract

Despite the popular belief in the anti-tumor properties of *Vespa bicolor* venom (VBV), there is limited scientific evidence to support this claim. This study is the first to examine the anti-tumor effects of VBV on liver cancer, both alone and in combination with cisplatin (DDP), through in vitro and in vivo experiments. In vitro experiments evaluated VBV and its combination with DDP on HepG2 cell proliferation, invasion, migration, and apoptosis. Animal studies examined the tumor-suppressive effects, safety (hepatotoxicity and nephrotoxicity), and immune impact of these treatments in tumor-bearing mice. VBV monotherapy significantly inhibited the growth of HepG2 cells by suppressing their proliferation and invasion and induced apoptosis in vitro. Notably, low VBV concentrations significantly promoted the proliferation of normal liver cells (L-02), suggesting a hepatoprotective effect. In vivo, VBV monotherapy enhanced immune function and exhibited tumor suppression comparable to DDP monotherapy but did not induce significant liver or kidney damage. In addition, VBV combined with DDP synergistically enhanced the anti-tumor effects of DDP, compensating for its limited apoptosis-inducing activity and insufficient enhancement of immune function. Initial studies have shown the strong potential of VBV as an anti-liver-tumor drug, highlighting its unique clinical value.

## 1. Introduction

Venom represents a crucial resource in the development of anti-tumor therapeutics [1,2], exemplified by honeybee venom, which has been the subject of extensive scientific investigation. Studies have demonstrated that honeybee venom exhibits notable anti-tumor activity and comprises a range of bioactive compounds with anti-tumor properties [3,4,5]. In recent years, breakthroughs in the cultivation technology of *Vespa bicolor* Fabricius, a wasp found in South China, have sparked widespread academic interest. The venom of *V. bicolor* exhibits multiple biological activities such as anti-inflammatory, anti-aging, and antibacterial activities, along with in vitro toxicity to liver tumor cells [6,7]. Additionally, it has been used to treat rheumatism, and in Chinese folk medicine, practitioners have combined the sting of *V. bicolor* with traditional Chinese medicine’s acupoint therapy for cancer treatment, yielding promising results [8]. These findings highlight the potential clinical significance of *V. bicolor* venom (VBV).

Vespa venom contains complex active components secreted by the venom glands. Previous studies on VBV have identified two antibacterial peptides (MP-VBs and VESP-VBs), a serine protease inhibitor (bicolin), and a smooth-muscle-contracting peptide (Vespin-BF) [9,10,11]. However, Wu et al. identified that the primary components of VBV from the wasp in South China included (1) peptide classes such as mastoparan-like peptide (Vb-MLP 12a); (2) protein types including hyaluronidase A, phospholipase A1 (two types), and antigen 5 protein; and (3) amines such as serotonin (5-HT) [7].

The mastoparan peptide family in Vespa venom is a key molecule in inducing toxicity by triggering allergic and inflammatory reactions [12,13,14,15,16]. Additionally, mastoparan exhibits significant cell membrane translocation activity and mitochondrial toxicity, and can induce cell apoptosis [17,18,19]. Different sources of mastoparan peptides show different anti-tumor activities; for example, mastoparan derived from *Vespula lewisii* shows significant inhibition of melanoma [20], whereas mastoparan from solitary wasp venom exhibits anti-glioblastoma effects through membranolytic activity [21]. Furthermore, amidated mastoparan exhibits significant inhibitory effects on leukemia, myeloma, breast cancer, and multi-drug-resistant cells [22].

In terms of protein components, hyaluronidase A acts as a “diffusion factor”, promoting the distribution and absorption of active substances in the venom [23,24,25]. Phospholipase A1 hydrolyzes sn-1 fatty acids in phospholipids, generating free fatty acids and inducing local inflammation [26,27,28]. Antigen 5 is involved in various physiological processes, including cancer progression, reproduction, and allergic reactions [29,30]. Hyaluronidase A, phospholipase A1, and antigen 5 can trigger different levels of cross-reactivity, leading to allergic reactions and inflammation [31,32,33,34,35,36]. These proteins are regarded as the primary allergens in Vespa venom. Notably, acute inflammation and allergic reactions are believed to have an inhibitory effect on cancer [37,38,39,40,41]. For example, acute inflammation can promote the maturation of dendritic cells and initiate T-cell-mediated immune functions to suppress tumor development [42], whereas the elevation of IgE levels caused by allergic reactions can also enhance the body’s immune function to combat tumors [43,44,45].

Furthermore, 5-HT is the primary active antioxidant substance in Vespa venom [46], and as a neurotransmitter, it can influence bodily functions such as mood and sleep. 5-HT and its receptors are known to interact with the nervous system and immune cells [47,48], influencing cancer treatment by stimulating T cell proliferation; promoting B cell development, inducing DC maturation; and enhancing NK cell cytotoxicity [49]. In addition, 5-HT is thought to facilitate immune cell recruitment to inflammatory sites [49].

In conclusion, the various components of VBV derived from wasps in South China may have unique therapeutic effects in tumor treatment. However, there is a lack of experimental data supporting the overall effectiveness of VBV as a medicinal intervention (such as in apitherapy). Consequently, this study seeks to elucidate the effectiveness of VBV in the treatment of liver tumors. We collected VBV from South China and examined its inhibitory activity against liver cancer both in vivo and in vitro, either alone or in combination with the chemotherapeutic drug cisplatin (DDP), to provide a theoretical foundation for the application of VBV in tumor treatment.

## 2. Results

### 2.1. Quality Control of Venom

**Figure 1:**
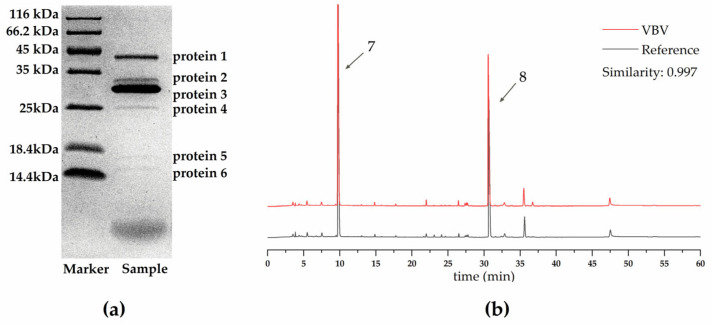
Quality evaluation of collected *Vespa bicolor* venom (VBV) samples, including (**a**) analysis of protein components by SDS-PAGE electrophoresis and (**b**) HPLC fingerprint analysis.

Using sodium dodecyl-polyacrylamide gel electrophoresis and high performance liquid chromatography fingerprint analysis, we detected six characteristic bands in the 10–45 kDa range (Figure 1a) in the collected VBV samples with an HPLC fingerprint similarity of 0.997 between the reference (Figure 1b), which met the quality standards reported in previous studies [50,51]. Moreover, prior research has established that proteins 1 and 4 correspond to hyaluronidase A and antigen 5, respectively, whereas proteins 2 and 3 represent the two subtypes of phospholipase A1. Within the HPLC fingerprint profile, peaks 7 and 8 are associated with 5-HT and the mastoparan-like peptide (Vb-MLP 12a), respectively [7].

### 2.2. Cell Viability Assay

**Figure 2:**
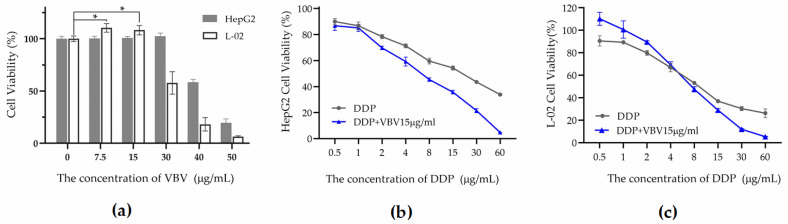
In vitro efficacy of VBV as a treatment for liver cancers either as a monotherapy or in conjunction with the chemotherapeutic drug cisplatin (DDP). (**a**) Cytotoxic effects of varying concentrations of VBV on HepG2 cells and L-02 cells. (**b**) Comparative analysis of the cytotoxicity of DDP alone versus its combination with 15 µg/mL VBV on HepG2 cells. (**c**) Comparative analysis of the cytotoxicity of DDP alone and in combination with 15 µg/mL VBV on L-02 cells. Data are expressed as means ± SD. Each experiment represents the mean values of six independent experiments. * *p* < 0.05 compared with negative controls.

To investigate the effects of VBV treatment, human hepatoma cells (HepG2) and normal liver cells (L-02) were evaluated after 24 h of treatment (Figure 2). VBV significantly inhibited the viability of both HepG2 and L-02 cells at concentrations ≥30 µg/mL. Notably, at VBV concentrations ≤15 µg/mL, no significant toxicity towards HepG2 cells was observed, while L-02 cells exhibited notable proliferative activity.

Furthermore, the anti-tumor efficacy of VBV (15 µg/mL) in combination with the chemotherapeutic agent DDP was evaluated. VBV enhanced the inhibitory effect of DDP on HepG2 cells at various concentrations (Figure 2b), resulting in a significant increase in the IC_50_ from 18.7 µg/mL to 6.1 µg/mL. Additionally, at lower concentrations of DDP (≤4 µg/mL), co-treatment with VBV mitigated the toxic effects associated with DDP on L-02 cells while promoting their proliferation (Figure 2c).

### 2.3. In Vitro Anti-Tumor Activity of VBV

**Figure 3:**
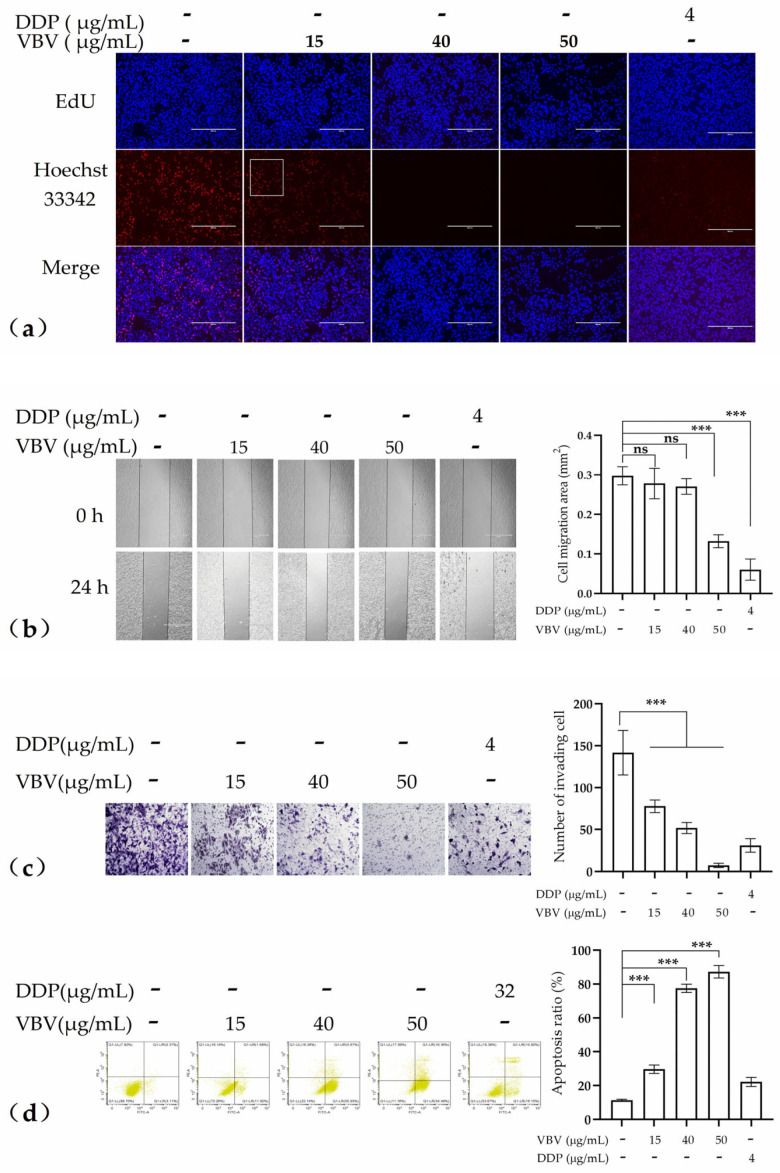
In vitro anti-tumor efficacy of VBV as a monotherapy against HepG2 cells. The effects of varying concentrations of VBV on HepG2 cells: (**a**) proliferation assessed using EdU, (**b**) migration assessed using scratch assays, (**c**) invasion measured using Transwell assays, and (**d**) apoptosis analyzed via flow cytometry. Data are expressed as means ± SD. Each experiment represents the mean values of three independent experiments. *** *p* < 0.001, significant difference; ns, no significant difference.

In this study, DDP was used as a positive control to investigate the inhibitory effects of varying concentrations of VBV (15, 40, and 50 μg/mL) on HepG2 cells. Initially, in the EdU cell proliferation assay, 15 μg/mL VBV was noted to have a significant inhibitory effect on HepG2 cell proliferation, whereas higher concentrations (40 and 50 μg/mL) completely suppressed this effect (Figure 3a). Subsequently, in the scratch assay, only the highest concentration (50 μg/mL) of VBV was observed to effectively impede HepG2 cell migration (Figure 3b). Furthermore, in the Transwell cell invasion assay, 15 μg/mL of VBV was observed to have a significant inhibitory effect on HepG2 cell invasion. However, higher concentrations (40 and 50 μg/mL) were unable to completely inhibit cell invasion (Figure 3c). Flow cytometric analysis revealed that all tested concentrations of VBV significantly induced early apoptosis in HepG2 cells and exhibited a strong concentration-independent effect.

### 2.4. In Vitro Anti-Tumor Activity of VBV in Combination with DDP

**Figure 4:**
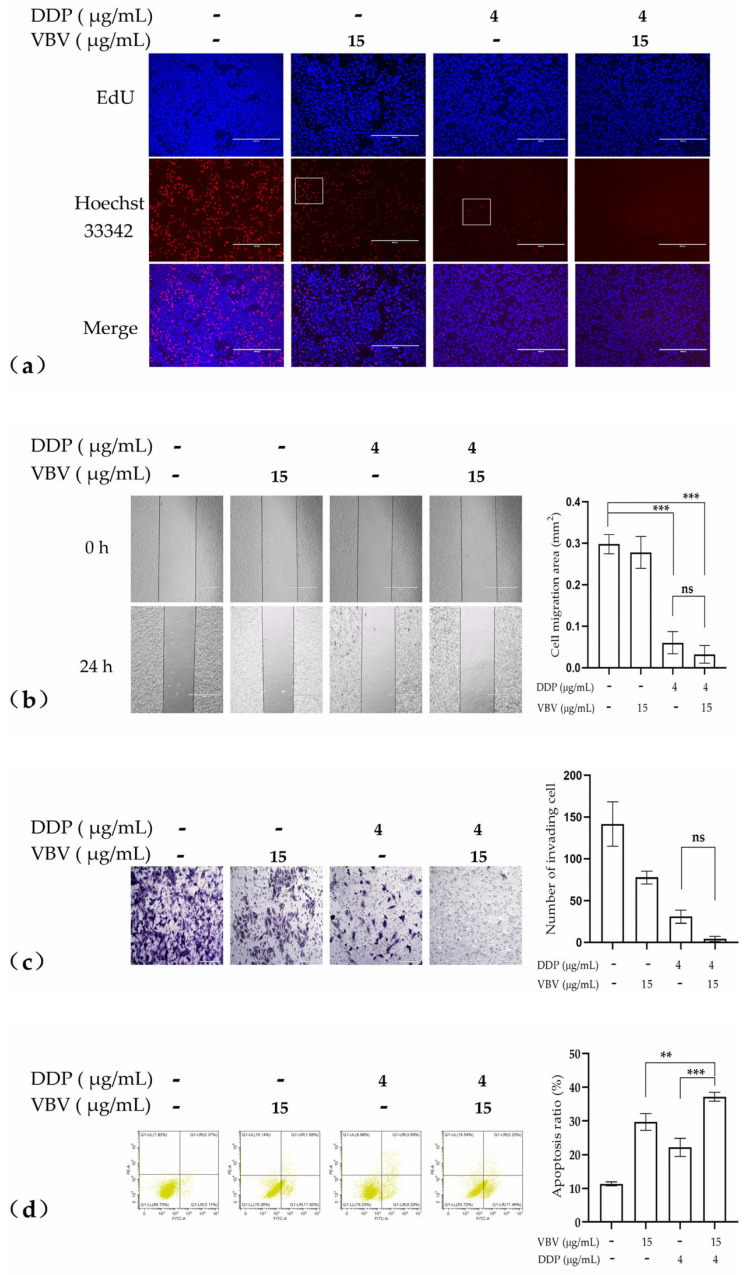
In vitro anti-tumor efficacy of combined with DDP. The effects of 15 µg/mL VBV combined with 4 µg/mL DDP on HepG2 cells: (**a**) proliferation assessed using EdU assays; (**b**) migration assessed using scratch assay; (**c**) invasion measured using Transwell assays; and (**d**) apoptosis analyzed via flow cytometry. Data are expressed as means ± SD. Each experiment represents the mean values of three independent experiments. ** *p* < 0.01, *** *p* < 0.001, significant difference; ns, no significant difference.

Based on the results of the cell viability tests, we further investigated the in vitro anti-tumor effects of VBV (15 μg/mL) in combination with DDP (4 μg/mL). The combined use of VBV and DDP markedly enhanced the inhibitory activity of DDP on HepG2 cell proliferation, migration, and invasion, resulting in nearly complete suppression, as demonstrated by EDU, scratch, and Transwell assays (Figure 4a–c). While the data did not reach statistical significance owing to the pronounced inhibitory effect of DDP alone (4 μg/mL), a notable enhancement in tumor suppression was observed with the combined administration of 15 µg/mL VBV and 4 µg/mL DDP. Flow cytometry revealed that VBV combined with DDP significantly enhanced the induction of apoptosis (Figure 4d). These results indicated the synergistic effect of VBV in combination with DDP.

### 2.5. In Vivo Anti-Tumor Effect of VBV Alone and in Combination with DDP

#### 2.5.1. Tumor Suppression Effect

**Figure 5:**
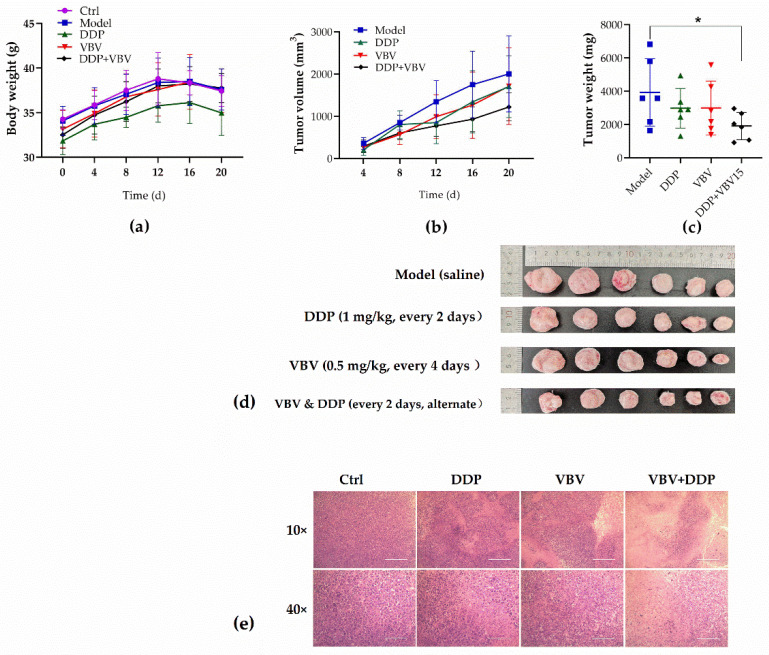
Tumor-suppressive effects of VBV alone and in combination with DDP in the murine model. (**a**) Weight changes of mice across different treatment groups over 20 days after tumor implantation. (**b**) Changes in tumor volume among the treatment groups. (**c**) Tumor weights in each group following 20 days of drug treatment. (**d**) Images of tumor tissue in each experimental group. (**e**) HE stained images of tumors in each experimental group. Data represent mean ± SD; n = 6 mice/group. * *p* < 0.05, significant difference.

Following the experimental procedure, the mice in each group exhibited uniform weight and satisfactory growth. After the 20-day drug administration period, all groups showed a consistent trend of weight gain, except for the mice in the DDP-treated group, which displayed slower weight gain (Figure 5a).

In terms of tumor inhibition, each drug treatment group exhibited varying degrees of tumor suppression compared to the model group (Figure 5b). The average tumor inhibition rates for the DDP and VBV monotherapy groups were approximately 24% and 23%, respectively, whereas it was approximately 51% for the combined treatment group, indicating a significant disparity (Figure 5c,d).

HE staining of the tumor tissues revealed that compared to the model group, both the DDP monotherapy and VBV monotherapy groups exhibited decreased tumor cell density, increased dead cells, and varying degrees of nuclear wrinkling and fragmentation. In the combination treatment group, a significant number of dead cells were observed, with a disorganized cell arrangement, nucleocytoplasmic concentration, nuclear shrinkage, and rupture (Figure 5e).

#### 2.5.2. Hepatorenal Toxicity and Immunomodulatory Effects

**Figure 6:**
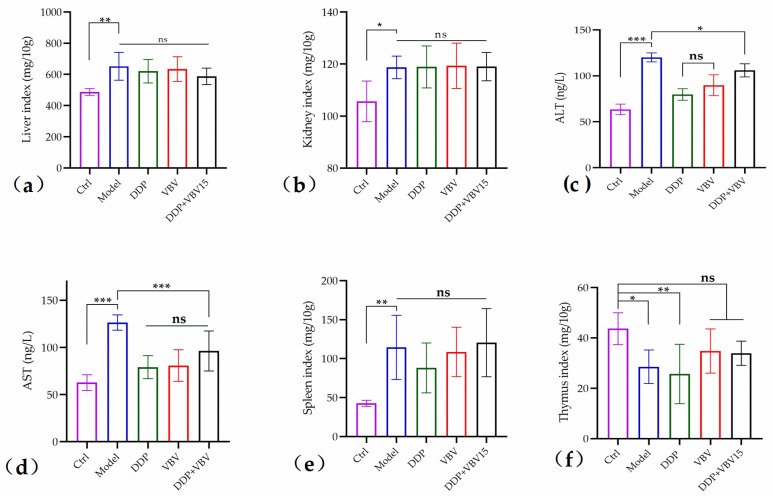
Hepatorenal toxicity and immunomodulatory effects of VBV alone and in combination with DDP evaluated in a murine model. Following 20 days of various drug treatments, we analyzed the differences in the following: (**a**) liver index, (**b**) kidney index, (**d**) spleen index, and (**e**) thymus index (**f**), as well as (**c**) ALT and (**d**) AST levels in serum among the different groups of mice. Data represent mean ± SD; n = 6 mice/group. * *p* < 0.05, ** *p* < 0.01, *** *p* < 0.001, significant difference; ns, no significant difference.

Regarding hepatic and renal toxicity, the liver and kidney indices in the model group were significantly elevated compared to the blank group. The kidney index of tumor-bearing mice across all groups remained relatively stable, with no significant alterations (Figure 6b). Conversely, the liver index after VBV or DDP treatment exhibited varying degrees of reduction; however, these changes were not statistically significant (Figure 6a).

Combined with the analysis of serum ALT and AST levels in mice (Figure 6c,d), the model group revealed significantly elevated ALT and AST indices, indicating that tumor growth induced substantial liver damage. VBV monotherapy exhibited similar effects as DDP monotherapy, both effectively mitigating liver injury during tumor progression. Furthermore, although combined administration caused a slight increase in the hepatic burden, it still significantly reduced liver damage compared to the model group.

In terms of immune function, compared to the blank group, mice bearing tumors exhibited a significantly enhanced spleen index but a significantly reduced thymus index (Figure 6e,f). DDP alone did not influence the effect of tumor growth on immune function; however, VBV alone or in combination with DDP significantly enhanced both peripheral and central immune organ indices, particularly restoring central immune function to normal levels.

## 3. Discussions

To explore the potential of VBV as a whole medicine (such as apitherapy) for tumor treatment, this study conducted the first in vivo and in vitro experiments to investigate its therapeutic effects on liver tumors when used alone or in combination with the chemotherapeutic drug DDP.

In vitro anti-tumor experiments revealed that 15 μg/mL of VBV exhibited significant ability to inhibit proliferation, suppress invasion, and induce apoptosis activity in HepG2 liver cancer cells. Notably, low concentrations of VBV (≤15 μg/mL) had a proliferative effect on normal liver L-02 cells, which contrasts with previous views on the hepatotoxicity of wasp venom [52]. This suggests that at low concentrations, VBV may exhibit potential hepatoprotective activity, offering new prospects for its future clinical applications. Furthermore, when DDP was used in combination with VBV at a concentration of 15 μg/mL, VBV exhibited a synergistic enhancement effect on DDP, particularly by compensating for the insufficient apoptosis-inducing activity of DDP. Combination therapy may have significant clinical implications and merits further investigation.

In the in vivo study, VBV (0.5 mg/kg every 4 days) exhibited a tumor-suppressive effect comparable to that of DDP (1 mg/kg every 2 days). Additionally, when used alone, VBV did not exhibit significant toxicity. The tumor suppression effect of VBV alone was not statistically significant, which could be attributed to the high density of tumor-bearing cells and the rapid growth rate of the tumor. Nevertheless, this highlights the strong feasibility of overall drug administration in tumor treatment. In addition, combined drug administration significantly enhanced the inhibitory effect of DDP on liver cancer, nearly doubling its efficacy, indicating the high potential of VBV for clinical application. Notably, the application of VBV, either alone or in combination therapy, can significantly enhance the immune function of tumor-bearing mice, particularly by restoring the thymus damage caused by tumor growth back to normal levels.

Although we did not conduct individual analyses of the VBV components, based on previous research and studies on allergies and inflammation [37,38,39,40,41], we infer that components such as hyaluronidase A, phospholipase A1, and antigen 5 protein, which are potent inducers of allergic reactions and acute inflammation, may, to some extent, serve as active ingredients that enhance immune function to combat tumors. Furthermore, the abundant mastoparan-like peptides (Vb-MLP 12a) in VBV may be the primary active substances responsible for inhibiting proliferation, suppressing invasion, and inducing apoptosis. Therefore, we speculate that there exists a synergistic enhancement effect among the various components of VBV in tumor therapy and that the application of VBV in tumor treatment is feasible.

Vespa and bee venom both offer significant potential for human medicinal research; however, the diverse species of *Vespa* and their complex breeding requirements compared to those of bees have hindered research efforts. Consequently, the development and application of Vespa venom lags far behind those of bee venom. With breakthroughs in *V. bicolor* breeding technology and the expansion of resources for VBV, further pharmacological activity and clinical application research on VBV are of great significance. This study highlights VBV as a highly valuable clinical treatment method comparable to bee therapy. While this study offers preliminary evidence suggesting the potential utility of VBV in cancer treatment, several limitations must be acknowledged, necessitating further research. Notably, the activities/toxicity profiles of individual components have yet to be thoroughly investigated and the complete composition of VBV remains unidentified. This study focused on the overall effects of VBV and provided theoretical support for its clinical application while also offering a reference for the direction of new drug development.

Overall, we have conducted the first preliminary exploration of the overall anti-tumor effects of VBV in both in vivo and in vitro settings. We believe that VBV has great potential as a novel anti-hepatoma drug with a unique clinical application value, whether used alone or in combination with DDP.

## 4. Conclusions

In conclusion, this study is the first to investigate the anti-tumor effects of VBV as a comprehensive treatment for liver tumors. Our findings demonstrate that VBV monotherapy can exert tumor-suppressing effects without causing liver or kidney damage, possibly through the inhibition of proliferation and invasion, induction of apoptosis, and enhancement of immune function. Notably, low VBV concentrations significantly promoted the proliferation of normal liver cells (L-02), suggesting a hepatoprotective effect. In addition, VBV combined with DDP synergistically enhanced the anti-tumor effects of DDP both in vivo and in vitro, compensating for its limited apoptosis-inducing activity and insufficient enhancement of immune function. Based on this, we conclude that the application of *V*. *bicolor* apitherapy in tumor treatment is feasible and may be another highly valuable clinical treatment method after bee apitherapy.

## 5. Materials and Methods

### 5.1. Venom

*V*. *bicolor* species were collected from the Guangdong province of China between July and November 2021. The *Vespa* species were identified by Professor Yun-Jiao Guo (Institute of Edible Insects, Dehong Normal University, Mangshi, Dehong Prefecture, Yunnan, China) as *V*. *bicolor* Fabricius. Crude venom was collected by electrical stimulation, dissolved in ultrapure water, and centrifuged at 3000× *g* rpm for 3 min. Subsequently, the supernatant was freeze-dried and stored at −80 °C. This study used previous research on the quality assessment of VBV to conduct protein and fingerprint analyses of the obtained samples [50,51].

### 5.2. Instrument and Regent

The following were used: high performance liquid chromatograph (LC-15C, Shimadzu, Kyoto, Japan); inverted microscope (EVOS FL, Thermo Fisher, Waltham, MA, USA); microplate reader (Synergy H1, Bio Tek, Winooski, VT, USA); flow cytometry (CytoFLEX S, Beckman Coulter, Brea, CA, USA); Dulbecco’s modified Eagle medium (Corning Inc., Corning, NY, USA); Roswell Park Memorial Institute (Buffalo, NY, USA)—1640, fetal bovine serum, penicillin, and streptomycin sulfate (Thermo Fisher); cisplatin (DDP, Beyotime, Shanghai, China).

### 5.3. In Vitro Anti-Tumor Assay

The HepG2 (human liver cancer cells, ATCC HB-8065) and L-02 (human normal hepatic cells, ATCC HL-7702) cell lines were cultured in Dulbecco’s modified Eagle medium or Roswell Park Memorial Institute—1640 supplemented with 10% fetal bovine serum, 100 IU/mL penicillin, and 100 µg/mL streptomycin sulfate, under a controlled environment of 37 °C with 5% CO_2_.

#### 5.3.1. Cell Viability Assay

After culturing HepG2 or L02 cells (5 × 10^3^ cells/plate) for 24 h in a 96-well plate, the cells were treated with sample solutions of VBV (100 µL) at varying concentrations (7.5, 15, 30, 40, and 50 µg/mL). The effects of DDP alone and in combination with VBV (15 µg/mL) were assessed. Additionally, groups treated with those without any sample solution and those devoid of cells served as negative and blank controls, respectively. Six parallel samples were prepared for each condition. Following a 24 h treatment period, cell viability was evaluated using the CCK8 kit (C0038, Beyotime), according to the manufacturer’s instructions.

#### 5.3.2. Analysis of VBV Anti-Tumor Effect

The anti-tumor effects of varying concentrations of VBV on HepG2 cells were assessed based on cell proliferation, migratory activity, invasion, and apoptosis. Cell proliferation and apoptosis experiments were conducted according to the manufacturer’s protocol using an EdU kit (C0078S, Beyotime) and an Annexin V-FITC/PI kit (C1062M, Beyotime), respectively. The ability of VBV to inhibit HepG2 cell migration and invasion was evaluated using scratch and Transwell assays. The scratch wound assay was conducted by scratching a straight line in the middle of HepG2 cells (5 × 10^4^ cells/plate) cultured for 24 h in a 6-well plate. The Transwell assay was performed using 80 μL of Matrigel (Corning Inc.) in 24-well Transwell chambers containing 4 × 10^5^ cells per plate and cultured for 48 h. The tumor inhibitory effects of VBV at concentrations of 15, 40, and 50 μg/mL were investigated. In addition, the group treated with DDP alone and the group treated with the medium were used as the positive control and the blank, respectively.

#### 5.3.3. Analysis of the Anti-Tumor Effect of VBV in Combination with DDP

The experimental protocol for assessing the anti-tumor effects of DDP combined with VBV on cellular invasion, migration, apoptosis, and proliferation was conducted as described in Section 5.3.2. Additionally, the combination group (4 μg/mL DDP and 15 μg/mL VBV) was established for comparison with the monotherapy group (4 μg/mL DDP or 15 μg/mL VBV alone).

### 5.4. In Vivo Anti-Tumor Assay

Ethical clearance was obtained from the Ethics Committee of Guangdong Pharmaceutical University (Ethics No. gdpulacspf2017576). Thirty Kunming female mice (4–5 weeks) were randomly divided into five groups: blank group (Ctrl), model group, DDP monotherapy group, VBV monotherapy group, and combined treatment group. H22 cells (mouse hepatocarcinoma cells) from ascites cultures were collected, and 0.1 mL of this culture was injected subcutaneously into the right axilla of the mice in each group (other than the blank group). After 4 days, small tumor clumps were observed, and treatment was initiated in each group.

DDP and VBV were formulated in isotonic saline solutions at 1 mg/kg and 0.5 mg/kg, respectively. DDP and VBV monotherapies were administered intraperitoneally every 2 and 4 days, respectively. The combined treatment group (VBV+DDP) was administered 1 mg/kg of DDP alone (first) or 0.5 mg/kg of VBV alone every two days, alternating between the two treatments.

In addition, the blank and model groups were injected with 0.1 mL isotonic saline solution every 4 days. The animals were treated and observed for 20 days, and the weight of the mice and tumor volume were measured every 4 days. Following the final dose (euthanized by exsanguination), blood, tumor tissues, and organs were collected for subsequent analyses, including plasma ALT/AST levels, visceral index, HE staining of tumor sections, and tumor inhibition rate. AST, ALT, and HE tissue sections were entrusted to Guangzhou Huayun Biotechnology Co., Ltd. (Guangzhou, China). The weights (g) of the liver, kidney, spleen, and thymus were calculated using the following visceral index formula: weight (mg) of these organs divided by the body weight (10 g).

### 5.5. Statistical Analysis

Cell viability, proliferation, migration, invasion, and apoptosis assays were performed at least three times. All animals were included in all analyses, and representative results are shown. The statistical significance of the samples was analyzed using a one-way analysis of variance, and post hoc analyses were performed using Tukey’s HSD. The results are expressed as mean values ± standard deviation (SD). Statistical significance was set at * *p* < 0.05, ** *p* < 0.01, and *** *p* < 0.001.

## Data Availability

The original contributions presented in this study are included in the article. Further inquiries can be directed to the corresponding author(s).
